# Predicting deep well pump performance with machine learning methods during hydraulic head changes

**DOI:** 10.1016/j.heliyon.2024.e31505

**Published:** 2024-05-17

**Authors:** Nuri Orhan

**Affiliations:** Selçuk University, Faculty of Agriculture, Department of Agricultural Machinery and Technology Engineering, 42140, Konya, Turkiye

**Keywords:** Hydraulic head, Deep well pump, Machine learning, Groundwater level change

## Abstract

In this study, machine learning techniques were employed to estimate and predict the system efficiency of a pumping plant at various hydraulic head levels. The measured parameters, including flow rate, outlet pressure, drawdown, and power, were used for estimating the system efficiency. Two approaches, Approach-I and Approach-II, were utilized. Approach-I incorporated additional parameters such as hydraulic head, drawdown, flow, power, and outlet pressure, while Approach-II focused solely on hydraulic head, outlet pressure, and power. Seven machine learning algorithms were employed to model and predict the efficiency of the pumping plant.

The decrease in the hydraulic head by 125 cm resulted in a reduction in the pump system efficiency by 6.45 %, 8.94 %, and 13.8 % at flow rates of 40, 50, and 60 m^3^ h^−1^, respectively. Among the algorithms used in Approach-I, the artificial neural network, support vector machine regression, and lasso regression exhibited the highest performance, with R^2^ values of 0.995, 0.987, and 0.985, respectively. The corresponding RMSE values for these algorithms were 0.13 %, 0.23 %, and 0.22 %, while the MAE values were 0.11 %, 0.2 %, and 0.32 %, and the MAPE values were 0.22 %, 0.5 %, and 0.46.% In Approach-II, the artificial neural network model once again demonstrated the best performance with an R^2^ value of 0.996, followed by the support vector machine regression (R^2^ = 0.988) and the decision tree regression (R^2^ = 0.981). Overall, the artificial neural network model proved to be the most effective in both approaches. These findings highlight the potential of machine learning techniques in predicting the efficiency of pumping plant systems.

## 1 Introduction

Water is an essential component of the Earth's ecosystem and a vital resource for sustaining life. The demand for water continues to rise due to population growth and improving living standards [1]. Concerning irrigation, water can be sourced from various bodies such as streams, lakes, and underground aquifers. Deep well irrigation, particularly in Turkey and the Konya region, is prevalent and extensively practiced. While only 4 % of Turkey's useable water is located in the Konya Closed Basin, this region accounts for 17 % of the country's irrigated agricultural land. Groundwater constitutes approximately 60 % of the water utilized in existing irrigated areas. As a consequence of this intensive usage, the groundwater level in the Konya Closed Basin has been steadily declining each year. Research conducted in the Konya Karapınar district since 1980 has revealed a water level drawdown rate of 0.7 m year^−1^ [2]. If the groundwater levels fall below a certain threshold, water extraction becomes economically unviable.

Groundwater levels experience fluctuations over time due to climatic factors and the utilization of deep well irrigation in agriculture. To counteract the declining static water levels in deep wells, manufacturers install pumps at greater depths or increase the depth of already installed pumps. The efficiency of a pumping plant varies depending on changes in the water levels within the deep wells. When the static water level in a well experiences drawdown, the hydraulic head of the pump also changes. Consequently, factors such as drawdown, flow rate, outlet pressure, and power consumption in a pumping plant can exhibit variations. These characteristics directly impact the efficiency of the pumping plant. However, a study conducted by Orhan et al. [3] on national deep well pumps revealed that an increase in the vertical hydraulic head leads to an improvement in the efficiency of the pumping system. In another study, it was found that reducing the static water load (hydraulic head) from 28 m to 14 m resulted in a decrease in the efficiency of the pumping system from 39.7 % to 35.8 % [4].

To evaluate the effectiveness of pumping plants, it is essential to evaluate metrics such as total head, flow rate, and power consumption. These characteristics experience annual fluctuations as static water levels undergo drawdown, resulting in a decrease in hydraulic head values. Monitoring techniques that are both time-consuming and costly must be employed to periodically inspect and determine the efficiency of pumping facilities. Electric motors utilized by pumps contribute to 22 % of the total energy consumption worldwide, with centrifugal pumps accounting for 16 % and rotodynamic pumps for 6 % [5]. By regularly monitoring and enhancing the efficiency of the installed pump systems, which consume a significant amount of electrical energy, operating costs can be reduced for the plant.

The operating and maintenance costs of a pumping plant constitute a significant portion, ranging from 92 % to 97 %, of the total expenditure [6]. According to Hovstadious [6], optimization techniques can lead to a reduction of approximately 10–20 % in pumping operating costs. Machine learning, widely applied across various domains, is commonly employed for solving optimization problems. Machine learning is a methodology that leverages existing data to draw inferences about previously unseen data [7]. In groundwater-related studies, machine learning methods have been extensively utilized for tasks such as groundwater level estimation [[8], [9], [10], [11]] and parameter estimation or optimization [[12], [13], [14], [15]]. Machine learning methods have also been applied to investigate pump performance [16]. However, the utilization of machine learning to evaluate the performance of deep well pumps at various water levels during or after a given period has not been explored.

This study aimed to construct models utilizing machine learning algorithms for predicting the system efficiency of a pumping plant at different hydraulic head values. The research involved measuring various parameters including the flow rate, outlet pressure, drawdown, and power consumption from the network, corresponding to different hydraulic head values of the pump. The calculated system efficiency was then used for analysis. Notably, there has been a lack of previous studies that have specifically applied machine learning algorithms to assess pump performance based on variations in hydraulic head. Therefore, this study represents a pioneering effort in analyzing pump performance by employing machine learning algorithms. The main novelty of this research lies in the development of models that enable the prediction and evaluation of pump performance at varying water levels, achieved by employing machine learning algorithms with distinct input parameters. This innovation is particularly significant as it allows for a comparison between input parameters, namely outlet pressure, power, and hydraulic head, which can be readily measured in the field, and the selection of two different input parameters within the context of machine learning algorithms.

## 2 Materials and methods

**Fig. 1:**
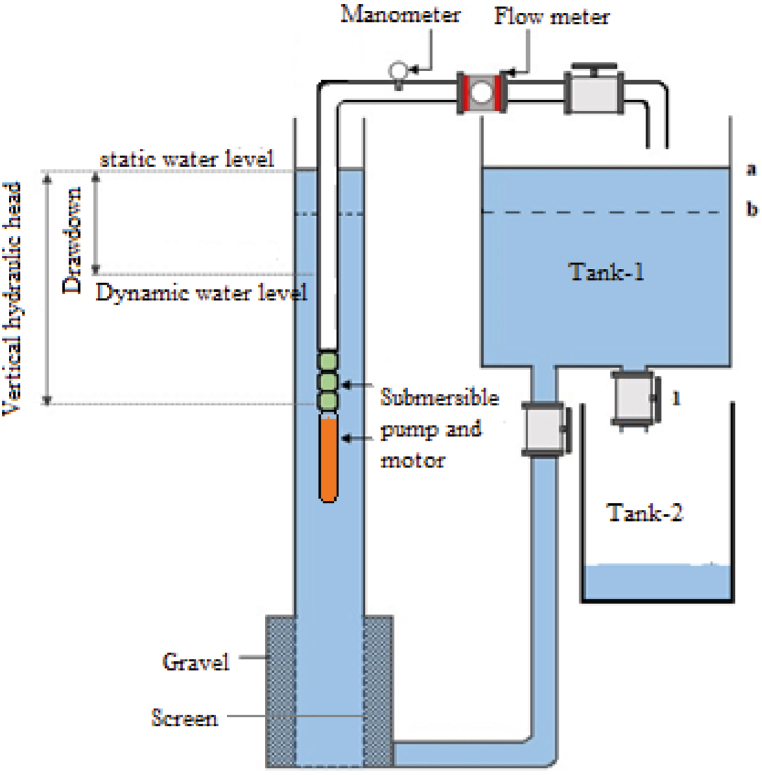
A schematic representation of the deep well test unit and well water heights.

This study was carried out at the Deep Well Pump Test Tower (Fig. 1), which was constructed under project number TUBITAK 213O140, at the Department of Agricultural Machinery and Technology Engineering within the Faculty of Agriculture at Selcuk University. For the experimental testing, a two-stage submersible pump with a 4-kW motor was employed. The elevation configurations implemented in the deep wells and a visual representation of the testing setup are depicted in Fig. 1.

Trial measurements were conducted using electromagnetic flow meters, power analyzers, digital manometers, temperature sensors, computers, and level meters. The technical details of the measuring instruments are presented in Table 1.

**Table 1:** Some technical characteristics of the measuring instruments.

Device	Technical specifications
Flow meter	S MAG 100 TİP, DN 80 flange connection electromagnetic flow meter, 220 V supplied digital indicator, instant flow, percent flow, total flow indicators. Adjustable 4–20 m/A plus and frequency output. Measurement error: 0.5 %.
Manometer	WİKA, 0–10 bar, Bottom installed, 4–20 m/A output.
Water level meter	Hydrotechnik brand, 010 type/1,5 V, 150 m scaled cable, voice and light indicator type.
Temperature sensors	Turck brand, 10–24 VDC, −50 … 100 0C, 4–20 mA output.
Computer	Asus intel core i7

During the experiments, various physical factors such as pump parameters, flow rate, outlet pressure, power consumption, and temperatures were meticulously recorded using a software and automation system. The measured values were wirelessly transmitted to the computer via a centralized data acquisition card using sensors that were compatible with the software and automation system, utilizing Bluetooth technology [3,17,18]. The experiments were carried out at six distinct vertical hydraulic head levels (2000, 1750, 1500, 1250, 1000, and 750 mm) and three different flow rates (40, 50, and 60 m^3^/h) at the standard operating speed of the pump. The well drawdown level (Δ), power consumption (N), and outlet pressure (Pb) were measured based on the predetermined parameters. After each flow rate, the pump was operated and the corresponding values were recorded before moving on to the next flow rate. While determining the pump's operating characteristics and performing calculations, relevant standards and data from previous studies were taken into consideration [[19], [20], [21]].

The vertical hydraulic head was determined by lowering the water level in tank 1 from level a to level b. Then, under controlled conditions (Fig. 1), the water in tank 1 was transferred to tank 2. Prior to altering the vertical hydraulic head, the pump was stopped. This methodology allowed for the determination of six different hydraulic load values.

Equations (1), (2), (3), (4) are used to calculate the system efficiency of the pumping plant [22].

(1) $$H_{m}=10,2*P_{b}+H_{d}+v^{2}/2\;g$$

(2) Q=A.v

(3) $$N_{h}=\left(Q*H_{m}*ɣ\right)/102$$

(4) $$\eta =\left(N_{h/}N_{ş}\right)\;*\;100$$

Here,

H_m_ indicates the total dynamic head (manometric) (m), P_b_ indicates the pump outlet pressure (bar), H_d_ indicates the dynamic water level (m), v indicates the velocity of water passing through the pipe (m s^−1^), g indicates acceleration due to gravity (m s^−2^), Q indicates the flow rate (m^3^ h^−1^), N_h_ indicates the hydraulic power at the pump outlet (kW), N_ş_ indicates the electrical power at the efficiency of the motor inlet (kW), and η indicates the pumping system. The pumping plant graphics were created using a trial version of Origin Pro 8.5.

### 2.1 Machine learning

**Fig. 2:**
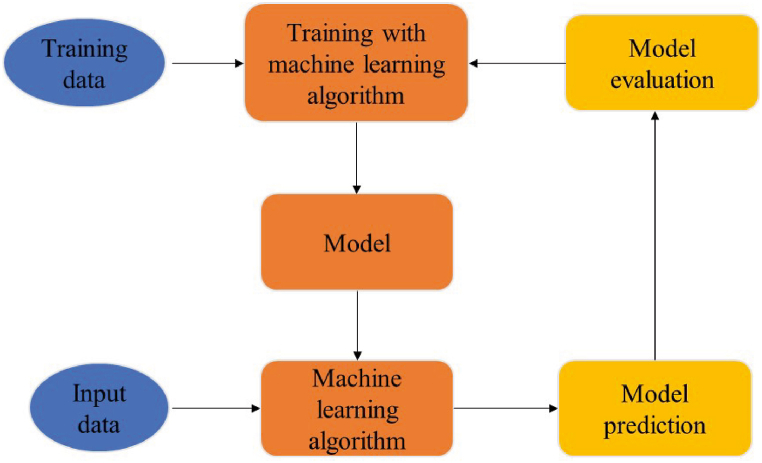
A schematic representation of the basic machine learning steps [25].

Machine learning empowers computers to autonomously solve problems by utilizing data and learning from it. It involves the creation of computer programs that can access data and learn from it without the need for explicit programming [23]. By leveraging machine learning, programs can find solutions based on the input and desired output, without the need for explicit instructions. Artificial intelligence, which is employed in machine learning, enables computers to learn from experience and improve their performance over time [24]. The basic machine learning process is illustrated in Fig. 2.

**Fig. 3:**
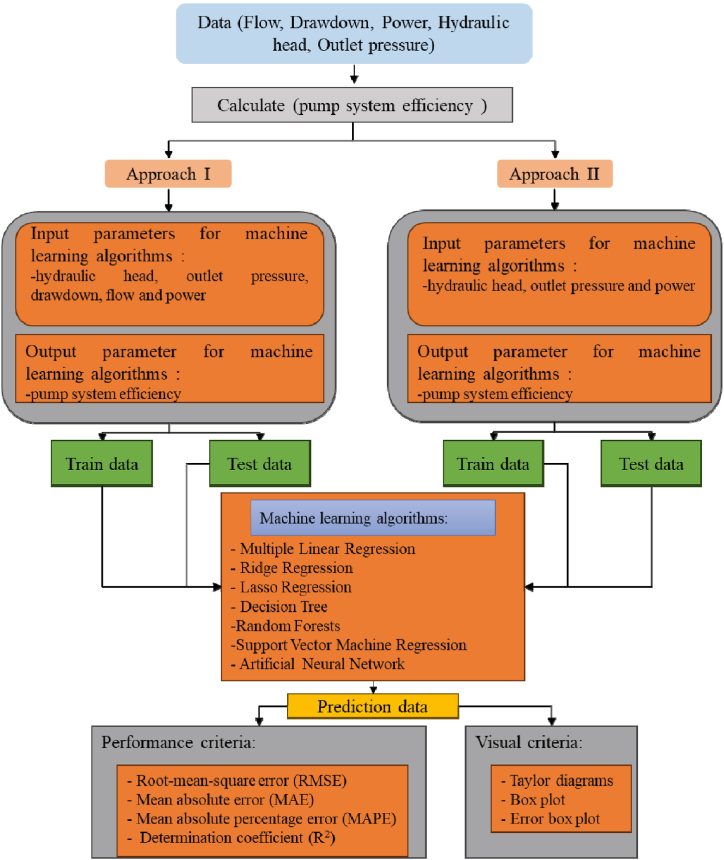
Flowchart of study.

In order to achieve accurate and efficient prediction of desired outcomes, various models have been developed with a high level of probability [25]. In this study, the main machine learning algorithms utilized are outlined below. These algorithms were implemented using the R statistics software [26]. The flow chart of the study is shown in Fig. 3.

#### 2.1.1 Multiple linear regression

The multiple linear regression model examines the overall relationship between the independent variables and the dependent variable. It assumes that there is no significant correlation among the independent variables (no multicollinearity), the residuals follow a normal distribution (normality), and the variances of the error terms are constant across the range of values for the independent variables (homoscedasticity) [27]. The multiple linear regression model takes the form in Equation (5).

(5) $$Y=\beta_{0}+\beta_{1}X_{1}+\beta_{2}X_{2}+\ldots +\beta_{p}X_{p}+\epsilon$$

Here, *Y* represents the response variable, *Xj* represents an independent variable, ***β****j* represents the coefficient, and *ɛ* represents the error term. The estimation of coefficients can be achieved by minimizing the loss function *L*, which is also referred to as residual sum of squares (RSS), as demonstrated in Equation (6).

(6) $$L=\sum_{i=1}^{n}{\left(y_{i}-{\hat{y}}_{i}\right)}^{2}=\sum_{i=1}^{n}{\left(y_{i}-{\hat{\beta }}_{0}-{\hat{\beta }}_{1}X_{i1}{-\hat{\beta }}_{2}X_{i2}-\ldots -{\hat{\beta }}_{p}X_{ip}\right)}^{2}$$

where ${\hat{y}}_{i}$ indicates the prediction for *y*_*i*_ based on the *i*th value of *X*. Therefore, *e*_*i*_ = *y*_*i*_ − ${\hat{y}}_{i}$ represents the *i*th residual.

#### 2.1.2 Ridge regression

Ridge regression is a popular refinement of conventional least squares linear regression that has gained traction in recent years. Unlike traditional least squares regression, ridge regression includes a regularization element, as described by la Tour et al. [28], which penalizes the loss function by incorporating an L2 regularization term. This regularization term minimizes the sum of the squares of the coefficients, as explained by Shahhosseini et al. [27]. In essence, ridge regression works to reduce the coefficients of impact of the model while avoiding overfitting, without enforcing coefficients to be zero. Thus, it is an effective way to minimize the impact of irrelevant features on the model, as noted by multiple studies in the field [29,30]. The loss function for the Ridge regression can be expressed by Equation (7).

(7) $$L=\sum_{i=1}^{n}{\left(y_{i}-\hat{y_{i}}\right)}^{2}+\lambda \sum_{j=1}^{p}\beta_{j}^{2}$$

Here, ***λ*** ≥ 0 represents a setting parameter. The value of ***λ*** must be determined separately by methods such as Cross Validation [28,31].

#### 2.1.3 Lasso regression

Lasso, as an extension of regularized linear regression, offers a unique approach by not only penalizing large values of the coefficients β but also setting them to zero if they are deemed irrelevant. Consequently, this approach may result in a reduced number of features included in the final model. The development of the Least Absolute Shrinkage and Selection Operator (LASSO) regression procedure was motivated by the need to address overfitting and simplify the linear regression model [32]. By adjusting the weights of the input parameters, the LASSO regression technique enhances the accuracy of the model by selecting relevant parameters and refining their adaptation. LASSO regression employs L1 regularization, which aims to minimize the absolute sum of the coefficients. This regularization method incorporates a ratio of the absolute values of the coefficients into the optimization process, allowing for better control over the influence of the parameters on the outcome [33]. The loss function for the LASSO regression can be expressed by Equation (8) [34].

(8) $$L=\sum_{i=1}^{n}{\left(y_{i}-\hat{y_{i}}\right)}^{2}+\lambda \sum_{j=1}^{p}{|\beta }_{j}|$$

#### 2.1.4 Decision tree

Decision trees, known as models for classification or regression, are structured in a tree-like format [35]. Each node in a decision tree represents a specific function and branches out into two or more groups for regression or classification samples. During the training phase, input variables are evaluated based on certain functions [36,37]. When dividing the dataset, there are multiple split points for each independent variable. The algorithm calculates the error between the predicted value and the actual values using a predefined fitness function at each split point. The variable with the lowest fitness function value is selected as the split point after comparing the split point errors among other variables [37].

#### 2.1.5 Random forests

A tree-based ensemble approach called bagging creates several trees by repeatedly resampling the training set and using the resulting data as new training sets [38]. A random forest is a collection of estimators with tree structures, where each tree depends on a random variable [39]. Because random forest breaks the association between trees, it is better than bagging.

A random *m* sample is selected from the *m* estimator as split candidates for creating the collection of preloaded trees from the original dataset while considering each split in a tree [40]. Additionally, out-of-bag observations from the original dataset that are not included in the bootstrap samples are considered when calculating the accuracy and error rates, which are then averaged (for regression) or voted for (for classification) across all observations [41].

#### 2.1.6 Support vector machine regression

The support vector machine (SVM) is a popular machine-learning technique for modeling the properties of the data and categorizing the data based on the properties [42]. SVM is a binary classifier that separates data samples along a linear separation hyperplane. Support vector regression, least squares SVM, and sequential projection algorithm SVM are commonly used [35]. The insensitive function and the Kernel function serve as the basic algorithmic building blocks of support vector regression (SVR) [43]. To achieve the nonlinear learning process in the original low-dimensional space, SVR functions transfer the data to a higher-dimensional feature space.

#### 2.1.7 Artificial neural network

The Neural Network is composed of a multi-layered structure, including the input layer, hidden layer, and output layer [44]. Various learning algorithms are commonly employed in artificial neural networks (ANNs), such as radial basis function networks, sensor algorithms, backpropagation, and flexible backpropagation [35]. By utilizing these learning algorithms to examine the relationship between input and output variables, the weights between each node in the neural network can be modified through a machine learning algorithm [42].

The neural network model involves two main steps. Firstly, the output value is calculated using a combination of input variables, hidden layer variables, the relationship between each variable (link and weight), and transfer functions. Secondly, in a process known as backpropagation, the discrepancy between the output value generated by the model and the actual value is utilized to ensure accurate computation and adjust the connections between variables. Each node in a neural network is assigned a weight based on the importance of its signal, and it employs input signals to compute information using an equation [42].

Prior to utilizing the artificial neural network model, the data was standardized. Several data normalization techniques are available, including the minimum rule, maximum rule, median, sigmoid, and Z-score [45]. The Z-score establishes a statistically normal distribution and indicates the position of each data point in terms of standard deviations. The standard value represents the distance of data points from the mean, which can be positive (above average) or negative (below average) [46]. In this study, the Z-score approach was employed to normalize the data in the artificial neural network models.

### 2.2 Model creation and performance

When dividing a dataset into a training set and a test set, a majority of the data is typically used for training, while only a small portion is reserved for testing. This division of the dataset is a crucial step in evaluating the model and assessing its performance. The most reliable approach to evaluate the accuracy of the model is to test its performance on the withheld data. Generally, 70 % of the data is allocated for training, while the remaining 30 % is used for testing. Assessing the model's performance using a dataset that was not used for training provides a more effective means of determining its accuracy [47].

Various error metrics are employed to evaluate the performance of prediction models and measure the degree of proximity between the predicted values of the model and the actual values [34]. Different metrics are commonly used to assess the performance of machine learning models. In this study, the root mean square error (RMSE) and mean absolute error (MAE) were utilized as measures of the differences between the predicted and observed values [48]. The performance evaluation was based on the R^2^ value, which represents the percentage of variation in the response variable that can be explained by the independent variables [27]. A higher R^2^ value indicates a stronger explanatory relationship. Since RMSE and MAE are error measures, lower values of these metrics indicate better model performance [25,49]. For instance, if the RMSE value is zero, it indicates a very high level of model performance [25,50].

## 3 Results and discussion

**Fig. 4:**
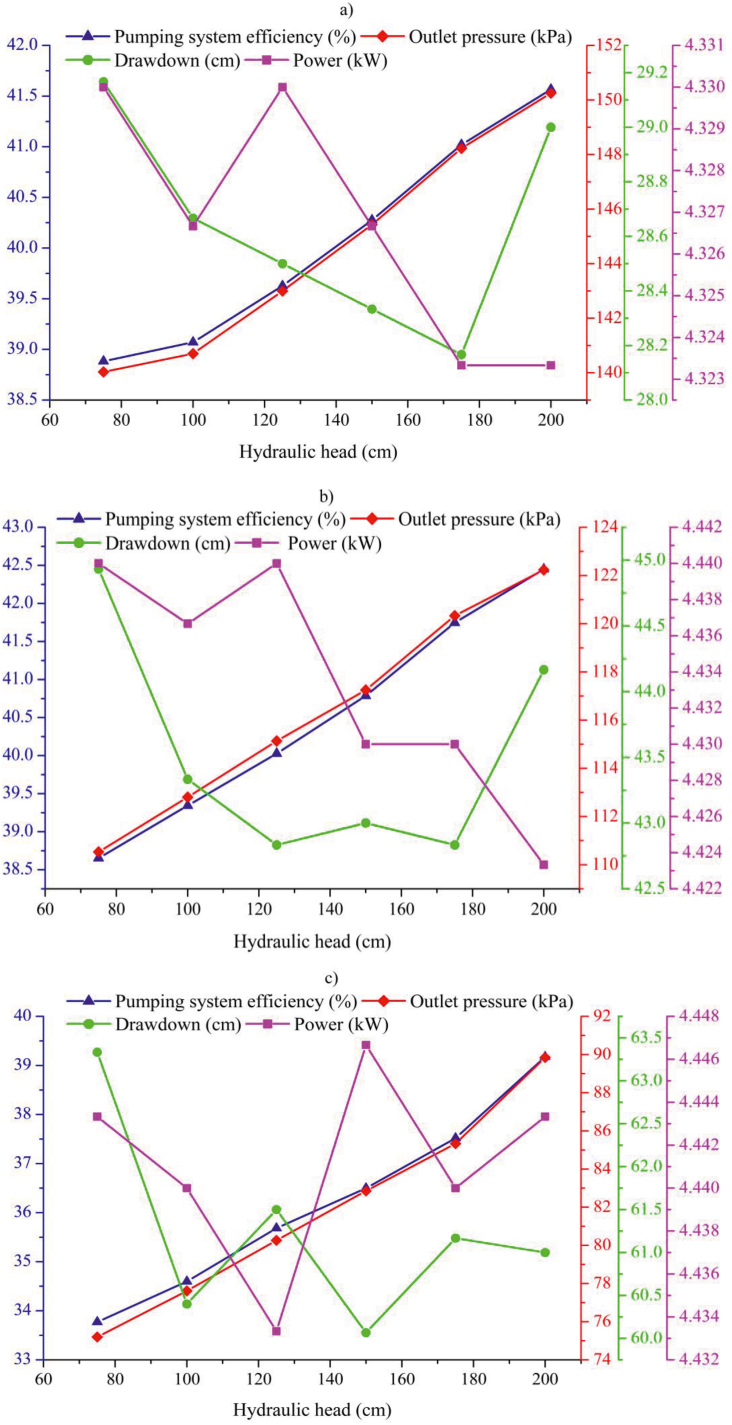
The changes in the measured parameters depending on the hydraulic head; (a) 40 m^3^ h^−1^, (b) 50 m^3^ h^−1^, and (c) 60 m^3^ h^−1^.

The data in this study were analyzed using two approaches: Approach-I and Approach-II. Approach-I involved the selection of the following variables for machine learning algorithms: hydraulic head, outlet pressure, drawdown, flow and power. In Approach-II, hydraulic head, outlet pressure, and power were chosen as variables since they were more easily monitored in the field. A total of 54 data points were collected for each variable. Machine learning methods were applied to develop models using these variables. The variations in the measured data related to the hydraulic head in the pumping plant are depicted in Fig. 4.

As the hydraulic head decreased across all three flow rates, both the pump system efficiency and outlet pressure values also decreased. Specifically, a decrease of 125 cm in the hydraulic head resulted in a reduction of 6.45 %, 8.94 %, and 13.8 % in pump system efficiency at flow rates of 40 m^3^ h^−1^ (Figs. 4a), 50 m^3^ h^−1^ (Figs. 4b), and 60 m^3^ h^−1^ (Fig. 4c), respectively. Similar findings were reported by Odeh et al. [4], who observed that a 50 % decrease in static water load led to a 9.8 % decline in pumping system efficiency. Power values generally decreased with an increase in the hydraulic head. While drawdown values exhibited a decreasing trend with increasing hydraulic head, an increase was observed when the hydraulic head reached 200 cm. Orhan et al. [3] obtained similar results in their study on a vertical shaft deep well pump.

### 3.1 Comparison of machine learning algorithms applied in approach-I

The application of the *t*-test in the analysis of linear regression is presented in Table 2. The model achieved a high level of explanatory power, as indicated by an Adj.R^2^ value of 0.994. Additionally, the residual standard error, which measures the dispersion of the observed data points around the fitted values, was determined to be 0.1907. Estimated coefficients of the variables in the model, along with their corresponding t-values, are provided in Table 2. Notably, with the exception of the drawdown variable, all t-values demonstrate statistical significance, suggesting a significant relationship between these variables and the outcome of the model.

**Table 2:** Approach I linear regression model results.

	Estimate	Std. Error	t-value	p-value
Intercept	−14.795	5.95	−24.84	2 × 10^−16^**
Flow	1.65	0.15	10.5	1.16 × 10^−12^**
Drawdown	−0.03	0.03	−0.92	0.36
Power	9.43	2.97	3.17	0.003**
Hydraulic head	−0.025	0.004	−5.84	1 × 10^−6^**
Outlet pressure	0.58	0.041	14.12	2 × 10^−16^**

**Fig. 5:**
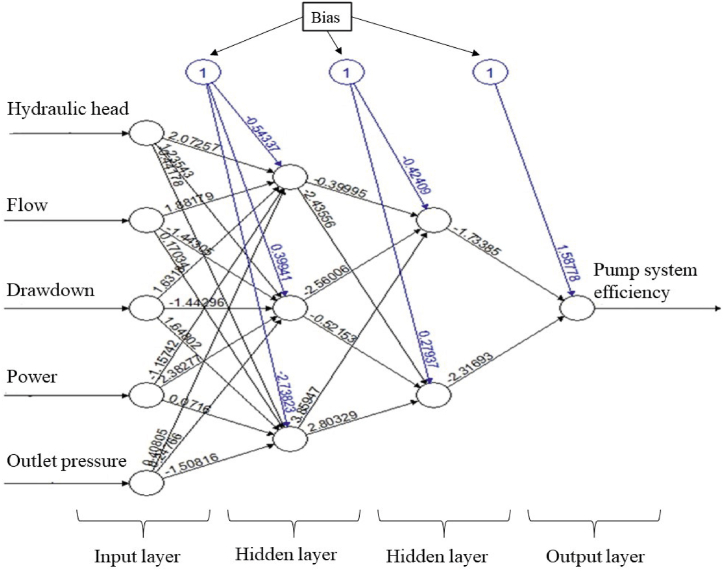
The artificial neural network in approach-I.

The artificial neural network model is visualized in Fig. 5. While constructing this model, two hidden layers were created. The first hidden layer had three neurons, and the second hidden layer had two neurons. The threshold value was 0.04, and the learning rate was 0.05. The model was trained in 209 steps. A widely used feedforward backpropagation network was chosen for system modeling [51]. In this study, the feedforward backpropagation algorithm is used. In regression problems model training, linear.output = TRUE is assigned. In addition, the loss function is assigned as SSE in the study.

The support vector machine regression algorithm was built with parameters SVM-Type: eps-regression, SVM-Kernel: radial, cost: 1, gamma: 0.2, and epsilon: 0.1. In this case, the number of support vectors was 13, and the value of R^2^ was 0.987. In the SVM model, the best estimation result was given when the Kernel function was assigned radial.

**Fig. 6:**
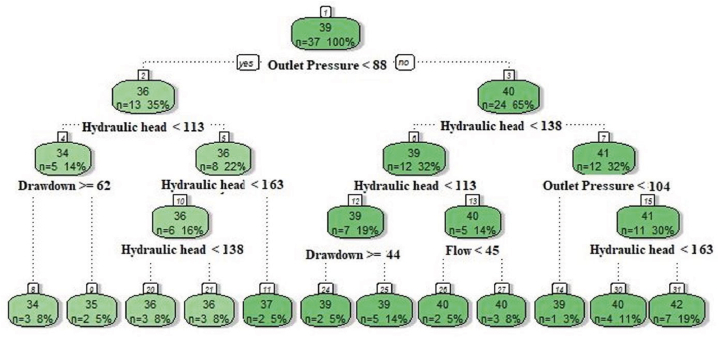
The decision tree model developed based on approach-I.

With the following settings, a decision tree regression was created: minsplit: 5, cp: 0, and maxdepth: 4. The tree model developed for the decision tree method is shown in Fig. 6. There were 12 terminal nodes, 10 decision nodes, and one root node in the decision tree. The rule was presented at the decision node with the highest hydraulic head value (6 numbers). Two drawdown rules, one pressure rule, and one flow rule were provided in the decision tree. The decision rule started with 88 kPa of pressure on the left and >88 kPa of pressure on the right of the decision tree.

Cross-validation was performed for the optimal lambda in ridge and lasso regression. The optimal λ values for the data in Approach-I were 0.142 for ridge regression and 0.011 for lasso regression.

**Fig. 7:**
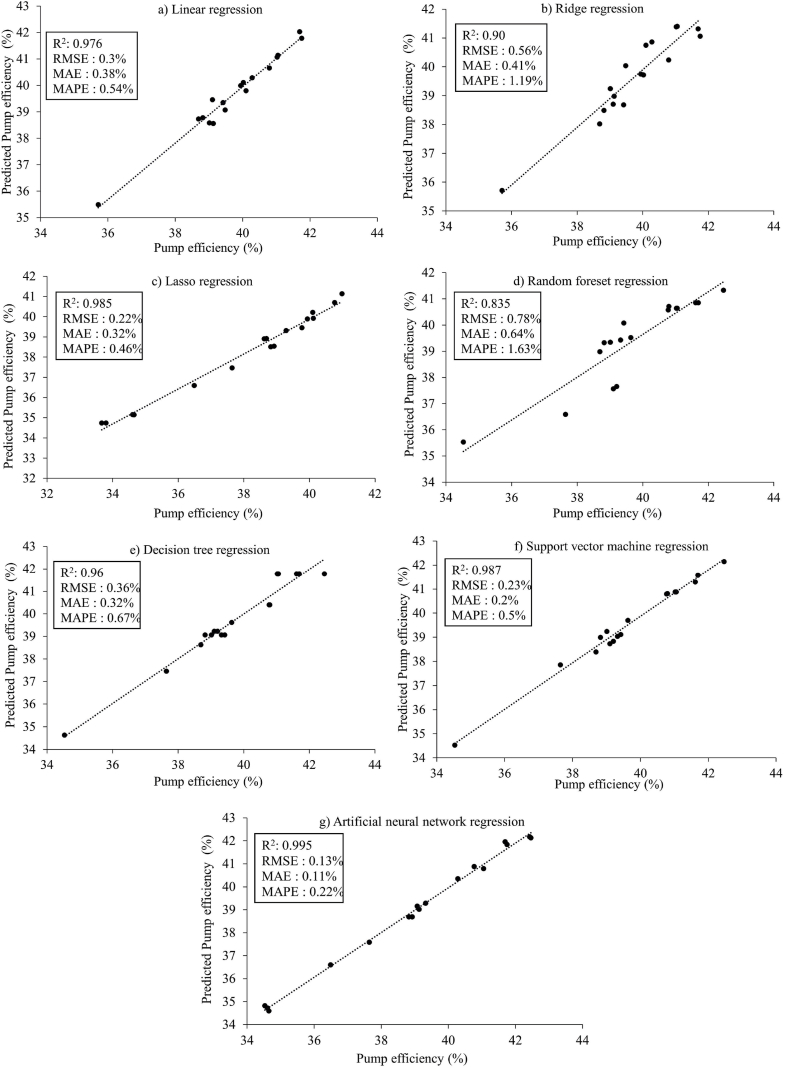
Scatter plots illustrating the models for Approach I.

**Fig. 8:**
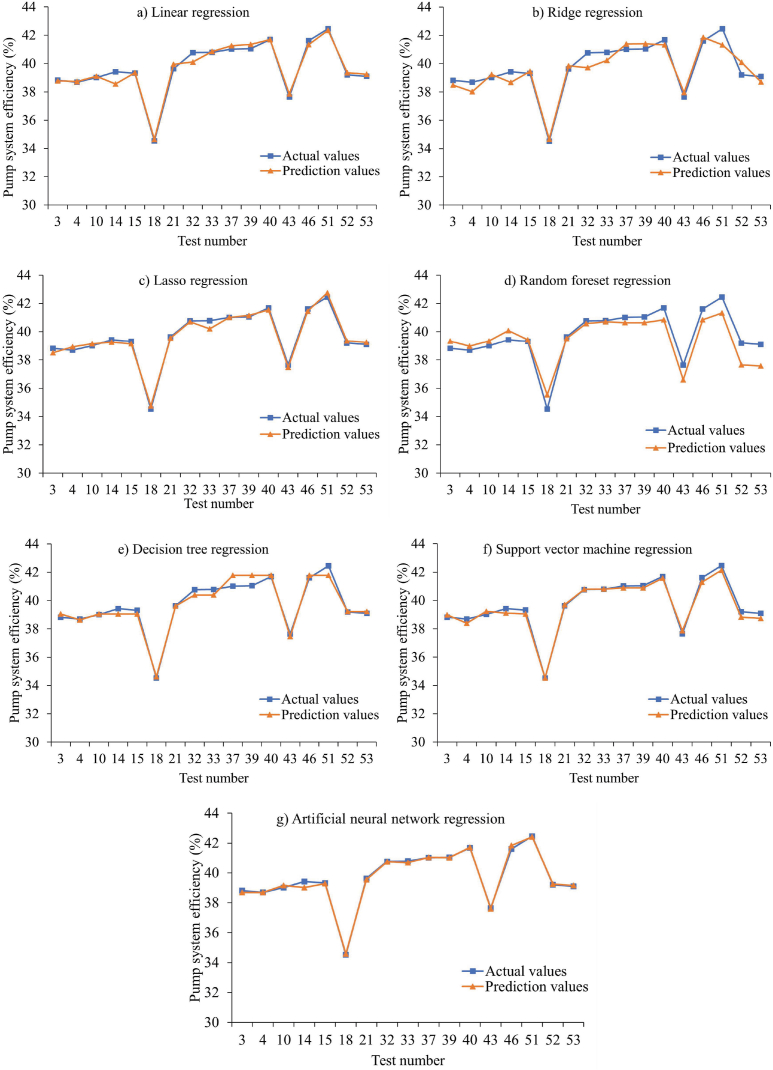
A comparison of the efficiency of the estimated and actual pumping systems based on the data used in Approach-I.

Fig. 7 displays the scatter plots and prediction performances of the machine learning algorithms applied in Approach I. Among the three models with the best performance, the artificial neural network model achieved an R^2^ value of 0.995, making it the top-performing model. It was followed by the support vector machine regression model with an R^2^ value of 0.987 and the lasso regression model with an R^2^ value of 0.985. On the other hand, the random forest regression model showed the lowest performance with an R^2^ value of 0.835. The linear regression, decision tree regression, and ridge regression models had R^2^ values of 0.976, 0.960, and 0.901, respectively. When considering the RMSE, MAE, and MAPE values, the artificial neural network regression model outperformed the other models with error metrics of 0.13 %, 0.11 %, and 0.22 %, respectively. Following the artificial neural network model, the support vector machine regression model showed the next best results with RMSE, MAE, and MAPE values of 0.232 %, 0.2 %, and 0.5 %, respectively. Although the lasso regression model ranked among the top three based on the R^2^ value, its performance was not as good when evaluated using the RMSE, MAE, and MAPE metrics. The higher error values observed in the random forest regression model could be attributed to its relatively poorer fit compared to the other models. Fig. 8 illustrates the comparisons between the predicted and actual pumping system efficiency using the machine learning methods in Approach I. The artificial neural network model demonstrated the best prediction results for the data in Approach I. Shin and Cho [42] also reported that the artificial neural network machine learning algorithm outperformed other algorithms when estimating heat pump efficiency. The neural network model offers several advantages over other machine learning models, including its ability to generalize data, process information efficiently, and extract crucial information for analysis and prediction [[52], [53], [54]]. Achieng [16] highlighted the accurate prediction of pump performance parameters by the support vector regression (SVR) model, emphasizing the high potential of machine learning in estimating pump efficiency.

### 3.2 Comparison of machine learning algorithms applied to the data in approach-II

Table 3 presents the utilization of the *t*-test within the framework of linear regression analysis. The examined model exhibits a considerable degree of explanatory capability, as evidenced by an Adj.R^2^ value of 0.976. Moreover, the residual standard error, which gauges the spread of observed data points around the estimated values, was found to be 0.4122. Estimated coefficients of the variables in the model, along with their respective t-values, are provided in Table 3. Notably, it is discerned that all variables exhibit statistically significant t-values, suggesting a significant association between these variables and the model's outcome.

**Table 3:** Approach II linear regression model results.

	Estimate	Std. Error	t-value	p-value
Intercept	−150.99	12.48	−12.1	1.12 × 10^−13^**
Power	39.01	2.72	14.29	1.08 × 10^−15^**
Hydraulic head	0.02	1.56 × 10^−3^	13.98	2.04 × 10^−15^**
Outlet pressure	0.133	5.16 × 10^−3^	25.8	2 × 10^−16^**

**Fig. 9:**
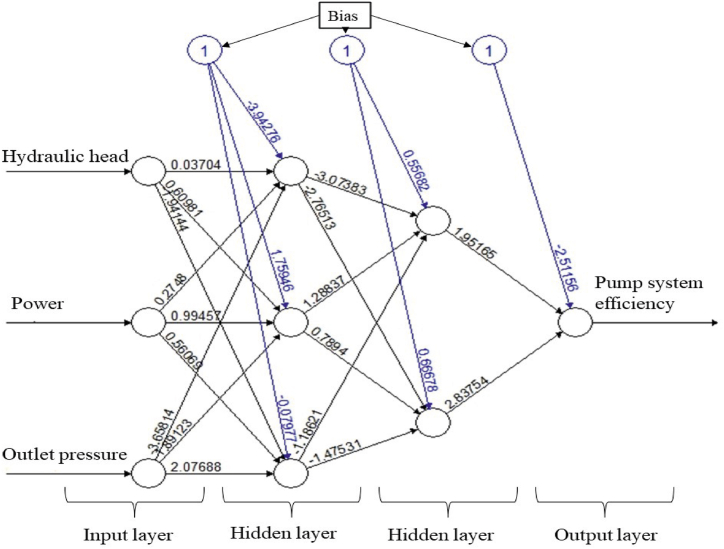
The neural network in approach-II.

The artificial neural network model is visualized in Fig. 9. The input parameters, hidden layers, and neuron counts of the data in approach-II were identical to those used to construct the data in approach-I. The constructed model was trained in 260 steps using this data.

The support vector machine regression algorithm was constructed using the following parameters: SVM-Type: eps-regression, SVM-Kernel: radial, cost: 1, gamma: 0.33, and epsilon: 0.1. In this particular approach, there were 12 support vectors, and the resulting R^2^ value was 0.988.

**Fig. 10:**
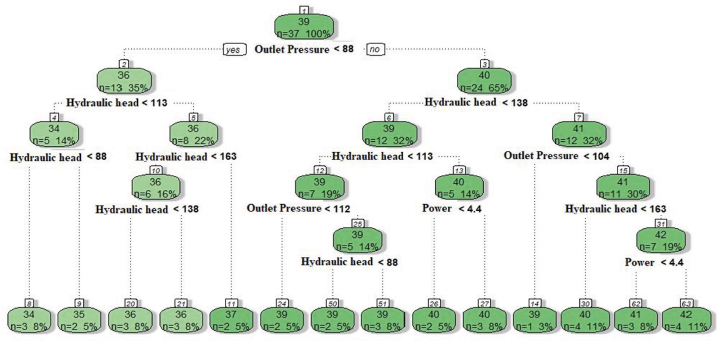
The decision tree model constructed for prediction based on approach-II.

A decision tree regression model was constructed using the parameters minsplit: 5, cp: 0.01, and maxdepth: 5. The resulting tree model developed through the decision tree method is depicted in Fig. 10. The decision tree consisted of 14 terminal nodes, 12 decision nodes, and 1 root node. The decision node with the highest hydraulic head value (node 8) presented the rule. Within the decision tree, there were two rules associated with power and two rules associated with outlet pressure. The decision rule began with <88 kPa of outlet pressure on the left side of the decision tree and >88 kPa of outlet pressure on the right side.

The optimal lambda values for the ridge and lasso regression models applied to the data in Approach II were determined as 0.142 and 0.01, respectively.

**Fig. 11:**
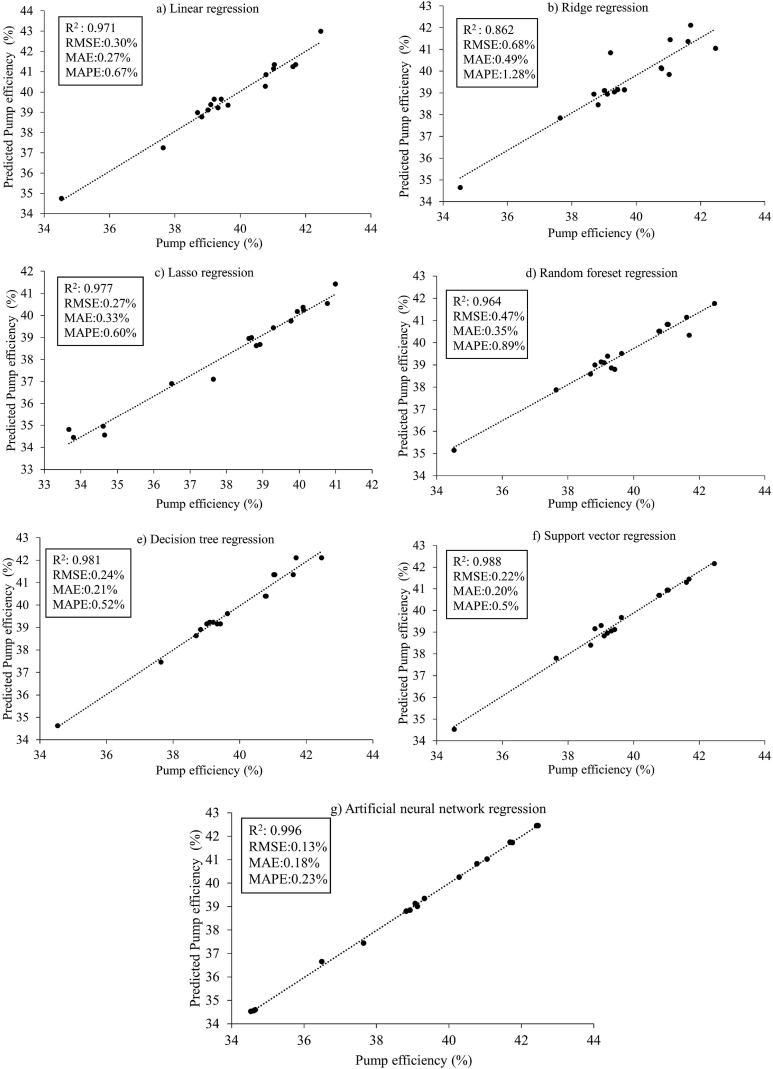
Scatter plots illustrating the models for Approach II.

The scatter plots and performance results of the machine learning algorithms for the data in Approach II are illustrated in Fig. 11. Similar to the findings in Approach I, the artificial neural network model (R^2^ = 0.996) exhibited the highest estimation accuracy for the data in Approach II. Following the artificial neural network model, the support vector machine regression (R^2^ = 0.988) and decision tree regression (R^2^ = 0.981) models demonstrated the best prediction performance. Conversely, the ridge regression model displayed the lowest performance with an R^2^ value of 0.862. The R^2^ values of the lasso regression, linear regression, and random forest regression models were 0.977, 0.971, and 0.964, respectively. Notably, while the lasso regression model ranked third in Approach I, it ranked fourth in Approach II.

**Fig. 12:**
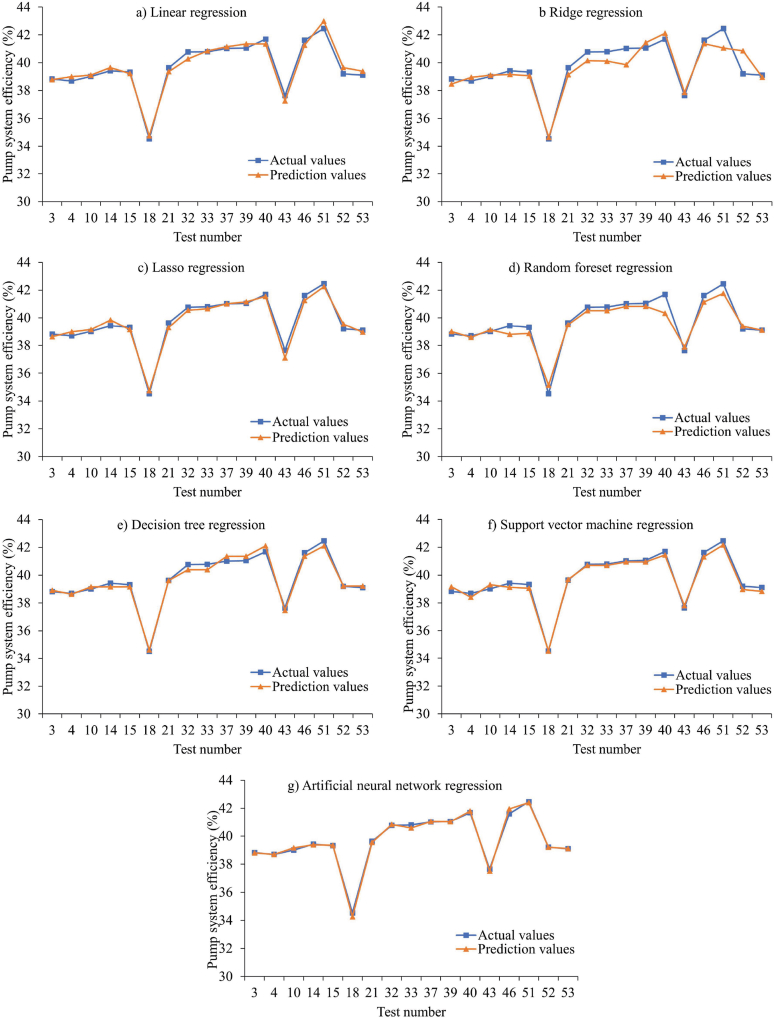
Comparison of the efficiency of the estimated and actual pumping systems for the data in approach-II.

Considering the RMSE, MAE, and MAPE metrics, the artificial neural network regression model yielded the best performance with values of 0.13 %, 0.18 %, and 0.23 %, respectively. Fig. 12 presents a comparison between the predicted and actual pumping system efficiencies for the machine learning methods implemented in Approach II.

**Fig. 13:**
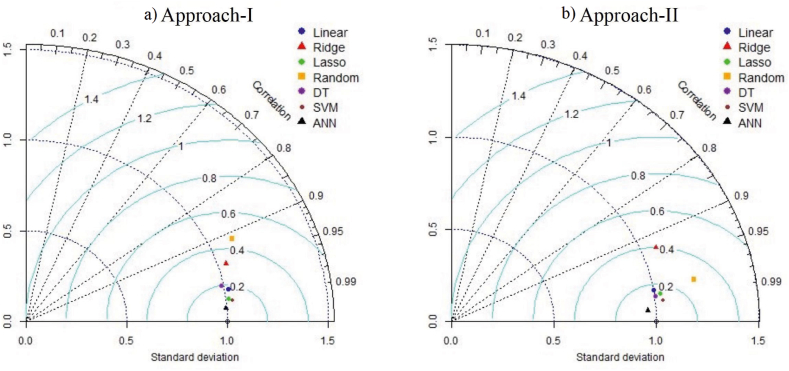
Taylor diagrams of Approach I (a) and Approach II (b) methods for each output variable (DT: Decision tree, SVM: support vector machine, ANN: artificial neural network).

In addition to R^2^, RMSE, MAE, and MAPE metrics, we conducted an analysis of the models' distribution using a Taylor diagram to assess their forecasting performance. The Taylor diagram was employed to evaluate the agreement between the reference data and the forecast data, as referenced by Bayram and Çıtakoğlu [55], Demir [56]. Fig. 13 displays the Taylor diagram for the models compared in Approach I and Approach II. In both approaches, the artificial neural network model exhibited the closest fit to the reference data. Following the artificial neural network, the support vector machine learning model demonstrated the closest resemblance to the reference values in both Approach I (Fig. 13a) and Approach II (Fig. 13b).

**Fig. 14:**
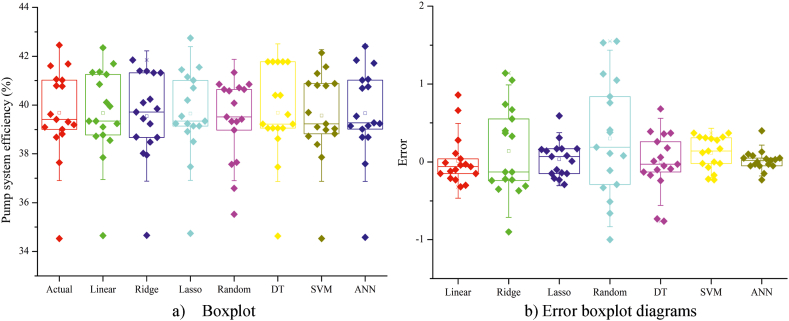
Boxplot (a) and error box diagrams (b) of Approach I models.

**Fig. 15:**
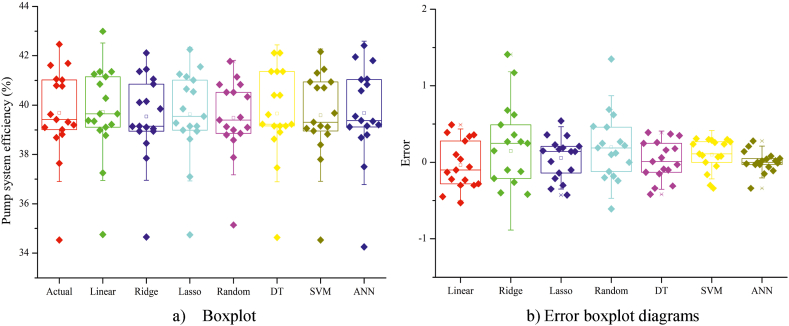
Boxplot (a) and error box diagrams (b) of Approach II models.

The boxplot and error box diagrams for the models in Approach I are presented in Fig. 14. It is clear from the boxplot diagram that the actual values and the values predicted by the artificial neural network (ANN) are highly similar (Fig. 14a). This similarity contributes to the difference and effectiveness of the error box diagram for the ANN model compared to the other models, as shown in Fig. 14b. In Approach II, similar results were obtained for the boxplot (Fig. 15a) and error box (Fig. 15b) diagrams generated for the models.

To compare the distribution of calculated and estimated efficiency values of the pump system, utilized the Kruskal-Wallis test (Table 4). This test assesses the null hypothesis that data from all groups come from a single population, employing a non-parametric rank-based test. Within the Kruskal-Wallis test, values from each group are ranked, and these ranks are used in lieu of the actual values to compute the test statistic. Previously, this test has been employed to compare the distribution of ML model predictions against measurements [[57], [58], [59]]. As observed in Table 4, the null hypothesis (H_0_) is rejected in the model predictions of pump system efficiency data. According to the results of the Kruskal-Wallis test, the predictions provided by these methods are found to originate from the same mean as the measured pump system efficiency values.

**Table 4:** Kruskal-Wallis test at 95 % confidence level.

Models	Pump system efficiency			
	Approach I		Approach II	
	p valus	H_0_	p valus	H_0_
Linear regression	0.207	Reject	0.18	Reject
Ridge regression	0.208	Reject	0.383	Reject
Lasso regression	0.162	Reject	0.359	Reject
Random foreset regression	0.633	Reject	0.169	Reject
Decision tree regression	0.155	Reject	0.15	Reject
Support vector machine regression	0.501	Reject	0.523	Reject
Artificial neural network	0.249	Reject	0.333	Reject

H_0_: There are differences between mean predicted and measurement values.

## 4 Conclusion

In this study, machine learning models were constructed to assess the efficiency of a deep well pumping plant by utilizing data that varied based on the vertical hydraulic head. The accuracy of the predicted performance was compared using linear regression, ridge regression, lasso regression, random forest regression, decision tree regression, support vector machine regression, and neural network regression models to estimate the system efficiency of the pumping plant.

The data in this study were divided into two approaches: Approach I and Approach II. Approach I included the following variables for machine learning algorithms: hydraulic head, outlet pressure, drawdown, flow, power, and outlet pressure. In Approach II, hydraulic head, outlet pressure, and power were selected as variables since they were easier to monitor in the field.

The top three performing machine learning algorithms applied to the input data in Approach I were neural network, support vector machine regression, and lasso regression, respectively. Similarly, in Approach II, the top three performing machine learning algorithms were artificial neural network, support vector machine regression, and decision tree regression. The neural network model showed the best performance for the data in both approaches. In the artificial neural network model, two hidden layers with three neurons in the first hidden layer and two neurons in the second hidden layer formed the most suitable model network for both approaches. The success of the neural network model can be attributed to its ability to generalize data and extract essential process information for analysis and prediction.

However, this study has certain limitations. Firstly, the calculation of system efficiency for the deep well pumping plant was solely conducted for six distinct hydraulic load values. Secondly, the investigation involved the comparison of various machine learning methods in the context of system efficiency estimation. Approach I examined input parameters such as hydraulic head, outlet pressure, drawdown, flow, power, and outlet pressure, while Approach II focused on hydraulic head, outlet pressure, and power. Based on the findings, it was determined that the data used in Approach II are sufficient to predict the efficiency of pumping plants under varying hydraulic head conditions.

The study demonstrated that machine learning techniques can be used to predict changes in the efficiency of the pumping system in the pumping plant due to variations in the deep well level. A method for estimating the variation in pumping plant performance, particularly when groundwater levels fluctuate annually, was developed. The data used in Approach II can effectively predict the performance of pumping systems during hydraulic head changes. By predicting a decrease in efficiency ranging from 0.5 % to 1 % in pumping facilities, inefficient working conditions can be avoided, and economic losses can be effectively prevented.

## Data availability statement

Author do not have permission to share data.

## CRediT authorship contribution statement

**Nuri Orhan:** Writing – review & editing, Writing – original draft, Visualization, Software, Resources, Methodology, Funding acquisition, Formal analysis, Data curation, Conceptualization.

## Declaration of competing interest

The authors declare that they have no known competing financial interests or personal relationships that could have appeared to influence the work reported in this paper.
